# An Inexpensive Open-Source Chamber for Controlled Hypoxia/Hyperoxia Exposure

**DOI:** 10.3389/fphys.2022.891005

**Published:** 2022-07-12

**Authors:** Tyler C. Hillman, Ryan Idnani, Christopher G. Wilson

**Affiliations:** ^1^ Lawrence D. Longo, MD Center for Perinatal Biology, Loma Linda, CA, United States; ^2^ Department of Bioengineering, College of Engineering, University of California, Berkeley, CA, United States; ^3^ Department of Pediatrics, School of Medicine, Loma Linda University Medical Center Loma Linda University, Loma Linda, CA, United States

**Keywords:** hypoxia, ischemia, neonate, stroke, inflammation, open-source

## Abstract

Understanding hypoxia/hyperoxia exposure requires either a high-altitude research facility or a chamber in which gas concentrations are precisely and reproducibly controlled. Hypoxia-induced conditions such as hypoxic-ischemic encephalopathy (HIE), obstructive or central apneas, and ischemic stroke present unique challenges for the development of models with acute or chronic hypoxia exposure. Many murine models exist to study these conditions; however, there are a variety of different hypoxia exposure protocols used across laboratories. Experimental equipment for hypoxia exposure typically includes flow regulators, nitrogen concentrators, and premix oxygen/nitrogen tanks. Commercial hypoxia/hyperoxia chambers with environmental monitoring are incredibly expensive and require proprietary software with subscription fees or highly expensive software licenses. Limitations exist in these systems as most are single animal systems and not designed for extended or intermittent hypoxia exposure. We have developed a simple hypoxia chamber with off-the-shelf components, and controlled by open-source software for continuous data acquisition of oxygen levels and other environmental factors (temperature, humidity, pressure, light, sound, etc.). Our chamber can accommodate up to two mouse cages and one rat cage at any oxygen level needed, when using a nitrogen concentrator or premixed oxygen/nitrogen tank with a flow regulator, but is also scalable. Our system uses a *Python*-based script to save data in a text file using modules from the sensor vendor. We utilized *Python* or *R* scripts for data analysis, and we have provided examples of data analysis scripts and acquired data for extended exposure periods (≤7 days). By using FLOS (Free-Libre and open-source) software and hardware, we have developed a low-cost and customizable system that can be used for a variety of exposure protocols. This hypoxia/hyperoxia exposure chamber allows for reproducible and transparent data acquisition and increased consistency with a high degree of customization for each experimenter’s needs.

## Introduction

Chronic and acute hypoxia exposure has been used in the past to study many different diseases, including hypoxic-ischemic encephalopathy (HIE) (Lacaille et al., 2019), intermittent sleep apnea (Toth and Bhargava, 2013; Arias-Cavieres et al., 2020; Lebek et al., 2020; Arias-Cavieres et al., 2021), ischemic stroke (Tsuji et al., 2013) and epilepsy (Toth and Bhargava, 2013; Lebek et al., 2020). Many studies use plethysmography (whole body and head-out) to assess short-term hypoxia and acquire other physiological data (Toth and Bhargava, 2013; Lebek et al., 2020). However, commercial plethysmography systems are comparatively expensive, typically require proprietary software for recording and monitoring pressure, volume, and flow, and may be difficult to set up without extensive technical support. Investigators focused on hyperoxia- or hypoxia-induced pathologies (neonatal HIE and stroke for example) often utilize simple hypoxia exposure systems that have rudimentary recording and environmental control (Yager, 2004). Experiments using hypoxia/hyperoxia exposure in animal models have used a variety of exposure times, oxygen concentrations, and other variables—contributing to high variability in neurological injury and making it difficult to determine the optimal hypoxia exposure settings for a given disease model. Because of this variability, there are significant challenges to reproducing experiments from different laboratories. In our laboratory, we have focused on HIE in preterm infants and have found it challenging to develop a reproducible model that will facilitate development of new HIE treatment, as have others (Rumajogee et al., 2016).

Our goal in this paper is to make our inexpensive, highly customizable open-source hypoxia chamber available to other investigators. Our chamber is useful across multiple experimental applications including: simulation of high altitudes, hypoxic ischemic stroke, sleep apnea, and other models requiring precise monitoring and control of the environment. Researchers studying HIE commonly use two types of exposures: long-chronic exposure or short-term hypoxia exposure (Vannucci and Vannucci, 2005; Lacaille et al., 2019). These exposures, however, also require animals’ homeostasis to be regulated by the use of external environment regulation. For example, the Rice-Vanucci Model pups undergo 10% oxygen exposure for one to 2 hours. They must also be kept at a constant temperature to reduce experimental variability (Vannucci and Vannucci, 2005). In Lacaille et al. (2019), long-term hypoxia requires monitoring and regulation to help stabilize the animals through a 7-day chronic hypoxia exposure (Lacaille et al., 2019). However, inconsistencies in factors other than oxygen level result in outcomes with a range of severity. To help reduce this variability in outcome, regulating and recording environmental variables continuously—such as temperature and humidity—can reduce data variability (Li et al., 2009; Summa et al., 2012). For example, the temperature should be monitored during hypoxia exposure as previous work shows that mice at 22°C ambient temperature had low plasma triglycerides (TG) and low-density lipoprotein cholesterol (LDL-C) levels that increased during intermittent hypoxia while mice at an ambient temperature of 30°C had high plasma TG and LDL-C levels that did not increase during intermittent hypoxia (Jun et al., 2013). Thus, these investigators concluded that ambient temperature affects lipid metabolism during hypoxia exposure (Jun et al., 2013).

Humidity is also an important variable to quantify when performing hypoxia testing. In moist air, mice’s metabolic rate was significantly lower than in dry air since evaporating the air’s moisture requires heat and would increase the energy needed for respiratory and metabolic processes (Phillips et al., 1950; Xiao et al., 2020). As a result, they found that, in dry air, less oxygen was required for respiratory and metabolic processes, which delayed the effects of hypoxia compared to in more humid air (Phillips et al., 1950). Additionally, others have found that heat stress and humidity can affect lactation in rodents (Xiao et al., 2020). Alterations to animal lactation further demonstrate the critical need to monitor and regulate temperature and humidity. The relationship between chamber oxygen pressure and arterial oxygen levels (Ogawa et al., 2019) still requires further investigation; however, barometric pressure may also play a role in batch variability.

Sound monitoring within the chamber is another key variable that should be monitored to minimize the possibility of hearing loss or sound-induced stress. Exposure to auditory stress during pregnancy in mice affected reabsorption and pup survival (Jafari et al., 2017). Likewise, light levels and day/night cycles should be monitored while the mice undergo intermittent or chronic hypoxia testing as, when combined with dim light at night, mice are more likely to exhibit anxiety and depression-like behavior (Aubrecht et al., 2013). Other studies have shown that, during hypoxia exposure, dams show depressive-like behaviors that are only observed in female rats (Kanekar et al., 2015). Thus if the mice are exposed to dim light at night during the hypoxia runs, other depression-like behaviors can serve as a confounding variable.

Monitoring oxygen levels is key for assuring reproducible hypoxia exposure, since the concentration of oxygen in the chamber must be reduced (Wenger et al., 2015) and tightly controlled. Wenger and others suggest that it can require hours to achieve the desired hypoxic conditions, but hypoxia levels can be rapidly altered by a brief opening of the hypoxia chamber to disrupt the chamber’s hypoxic conditions (Wenger et al., 2015). Others have found that exhaling breath with high levels of volatile organic compounds (VOC) occurs during reduced oxygen levels (Harshman et al., 2015). By ensuring that VOC concentration is similar across hypoxia runs, it is possible to ensure that hypoxic conditions do not vary between hypoxia exposures.

To reduce the cost and provide maximum customization in our chamber design, we have provided a detailed overview of our chamber and supporting software that relies upon inexpensive off-the-shelf components allowing investigators high-resolution monitoring and control over temperature, humidity, and oxygen levels without the need for proprietary software or custom fabricated equipment. We developed a system based on Free/Libre Open-Source (FLOS) principles so others can use the system for greater reproducibility experiments by labs of any size and funding status. By creating a system to monitor and modulate these environmental factors, we can unify these surgical models and their hypoxia exposures (Henderson et al., 2018). Our goal here is to create an inexpensive system for controlled gas exposure that will lower the bar of entry for other investigators interested in understanding physiological changes induced by hypoxia/hyperoxia.

## Methods

### Chamber Components

Exposure to hypoxia during a surgical procedure elicits a stress response from the animals exposed. Therefore, to reduce and quantify variability in environmental factors, we assembled a comprehensive set of tools to monitor environmental variables and maintain them within desired ranges for a given experiment. A complete list of the parts and components of the chamber are included in the Supplemental Material and Table 1. Additionally, the Python code, PCB file, 3D files and instructions for chamber assembly can be accessed on github.com (https://github.com/drcgw/hypox-chamber.git).

**TABLE 1:** Major components and prices included in the chamber creation as of June 2022.

Cage Aspect	Item		Description	Company	Part Number	Variable, Accuracy, Percision	Quantity Used	Quantity per Unit	Cost Per Unit
Cage Structure									
		Glass Tank	Aqueon Glass Aquarium Tank - 20 Gallon	PetCo	—	Chamber	1	1	$ 49.99
		Glass Tinting	One-way window film	Amazon	—	Visual Stimulus Reduction	1	1	$ 11.99
		Acrylic Sheet	Chamber top - 1/4 x 31.5 x 14.25 in	ACME Plastics	—	Chamber Lid	1	1	$ 45.00
		Junction Box	Steel Junction Box, 254 x 254 x 101.6 mm	Newark	40T7867	Electrical Input	1	1	$ 31.60
		Cable Management	3.35 inch black cable straps	Amazon	B0881FW8TQ	Electrical Management	Variable	50	$ 9.99
Airflow									
	Bulkhead		1/2 in female PVC bulkhead fitting	Amazon	—	Airflow - In	2	2	$ 12.49
	PVC Tubing		1/2 in FPT T joint	Home Depot	—	Airflow - In	3	1	$ 1 - 3
	—		1/2 in MPT Valve	Home Depot	—	Airflow - In	2	1	$ 1 - 3
	—		1/2 in MPT Adapters (1/4, 5 and 10 in)	Home Depot	—	Airflow - In	8	1	$ 1 - 3
	—		1/2 in MPT/FPT Elbow	Home Depot	—	Airflow - In	6	1	$ 1 - 3
	Brass Tubing		1/4 in Bulkhead	Amazon	—	Airflow - Out	2	2	$ 12.89
	—		1/4 in R-angle Elbow	Amazon	—	Airflow - Out	2	2	$ 14.59
	—		1/4 in Control Valve	Amazon	—	Airflow - Out	2	2	$ 14.99
Electrical									
	Sensor PCB*		Custom PCB boards for sensor attachment	OSH Park		Sensor Mount	5	5	$ 102.50
	Raspberry Pi**		Raspberry Pi 4B, 2 GB RAM	Adafruit	4292	Microprocessor	1	1	$ 45.00
	PiCamera		Raspberry Pi Camera 2	Adafruit	3099	Image (jpg) (8 megapixel)	1	1	$ 29.95
	Power Supply		Adjustable DC Powersupply 110 V - 220 V, Output: 0 - 24 V, 20 A 480 W	Amazon	B08GFSVHLS	Power (Percision: 0.1 V)	1	1	$ 35.99
	MCP9808		High accuracy I2°C Temperature Sensor	Adafruit	1782	Temperature (Accuracy: ± 0.25°C, Percision: ± 0.0625°C)	3	1	$ 4.95
	BH1750		Light Sensor I2°C	Adafruit	4681	Light (Lux)	2	1	$ 4.50
	BME280		Temperature, Humidity & Pressure sensor I2°C	Adafruit	2652	Temperature (± 1.0°C), Humidity (± 3%), Pressure (± 1.0 hPa)	2	1	$ 14.95
	CCS811		VOC and eCO2 Sensor	Adafruit	3566	CO2 & VOC	2	1	$ 19.95
	ADS1115		4 Channel 16 bit Analog to Digital Converter	Adafruit	1085	Converter (Percision 8-860 SPS)	1	1	$ 14.95
	Voltage Regulator		3.3 V 800 mA Voltage regulator	Adafruit	2165	Voltage (Accuracy: ± 1%)	5	1	$ 1.25
	0.1 uF Capacitor		0.1 uF capacitor, max of 50 V	Adafruit	753	Voltage (Accuracy: -20%/ + 80%)	5	10	$ 1.95
	10 uF Capacitor		10 uF capacitor, max 50°V low-fequency	Adafruit	2195	Voltage (± 20%)	5	10	$ 1.95
	Power Jack		Gravitech DC Power Connector 2.1 mm x 5.5 mm	Mouser	992-CON-SOCJ-2155	Voltage	5	1	$ 1.00
	D-Sub 15 Female		D-Sub High Density DBHD15, Female	Amazon	CNR15HDM-F-10PACK	Connector	8	10	$ 12.88
	VGA Cables		UGREEN VGA, 3 meter, male to male coaxial	Amazon	B00OZL3HLO	Cable	4	1	$ 7.99
	USB Cables		Micro-USB Cable, R-angle joint	Amazon	B09C5P7YFN	Cable	1	3	$ 9.99
	USB Cables		USB-C Cable, R-angle joint	Amazon	B09CG9LZSR	Cable	1	2	$ 9.99
	DC Power Cable		DC Power Pigtail, 3ft, Male 5.5 mm x 2.1 mm plug	Amazon	B08PYWN3T7	Cable	4	2	$ 9.99
	DC Cable Extender		6 ft DC Extention Cord, male to female	Amazon	B074WJZNZD	Cable	3	4	$ 13.99

* Can be replace with breadboards and jumper cables.

** Can also be run with Raspberry Pi 3B, 4B - 1 GB, 4B - 4 GB or 4B - 8 GB.

Additional hardware and custom parts can be found online at: https://github.com/drcgw/hypox-chamber.git and in the Supplemental Material.

### Systems Recording

**FIGURE 1:**
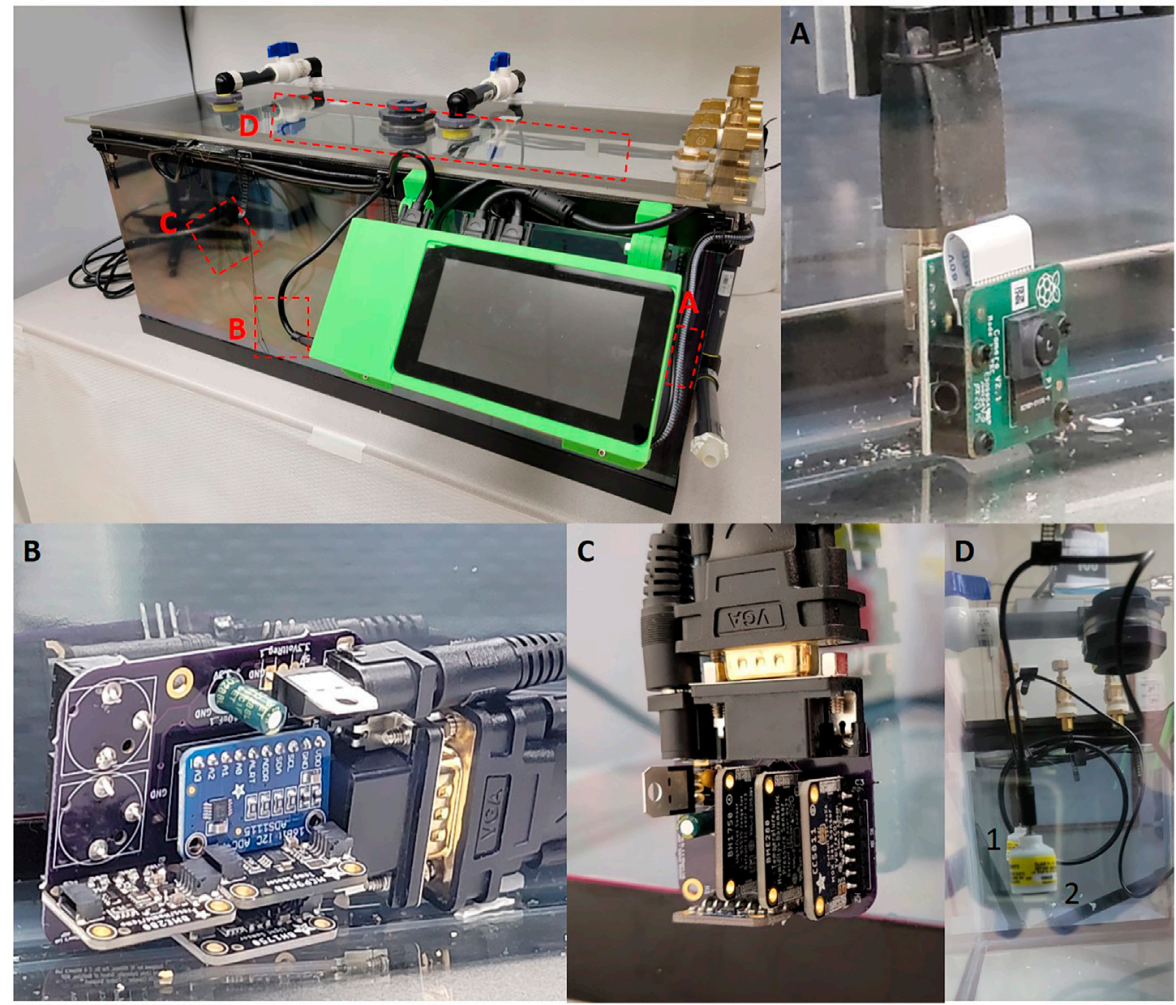
**(A)** Raspberry Pi Camera version two attached to the Raspberry Pi via a HDMI cable. **(B)** Central sensor PCB board with ADS1115, MCP9808, BME280 and BH1750 sensors, the rear of the board contains two Aux jacks which allow for Teledyne sensor attachment. **(C)** External sensor breakout which contains MCP9808, BME280, BH1750 and CCS811. **(D)** Teledyne oxygen sensors hung from the roof of the chamber utilizing cable management clips. Two sensors are held by cables clips on the sealing, identified by the one and two identifiers.

Temperature calibration was achieved by periodically taking temperature measurements with MCP9808, BME280, and/or BME680 sensors. Depending on the needs of the researcher we have designed the chamber to have some redundancy. The MCP9808 is a high precision (+0.0625°C) temperature sensor with a range between -40 and 125°C and accuracy of ±0.25 °C (Ada, 2021). In contrast the BME280 and BME680 are able to detect multiple variables including: temperature, humidity, pressure and total volatile organic compounds (Only BME680) but lose precision and accuracy. The accuracy and range of the BME280s sensors are: relative humidity ±3% between 0 and 100%, barometric pressure ±1 hPa between 300 and 1,100 hPa and temperature ±1.0 °C between -40 and 85 °C (Ada et al., 2015). The accuracy of the BME680s sensors are the same as the BME280 with the addition of a heated metal oxide resistor to look at total volatile organic compounds (tVOC) (Ada and Rembor, 2017). The BME280/680 sensors also have limitations regarding how many of each sensor type can be attached due to limited I2C addresses available for each sensor. The MCP9808 has eight possible addresses whereas the BME280 and BME680 only contain two I2C addresses which are duplicated for the two sensor boards (Ada and Montoya, 2017). For our chamber we utilize both MCP9808 and BME280 despite the overlapping temperature variable, we utilize the MCP9808’s precision to calibrate the BME280 however, utilization of any of these three sensors will also allow for complete data collection. After these measurements were taken, the PiCamera was programmed to take a picture of a mercury thermometer located within the chamber (Figure 1A). After collecting the data, these images were used to determine the actual temperature in the chamber. The actual temperature in the chamber was then compared with the sensor readings to determine the precision of the sensors and calibrate the temperature sensors accordingly. Using a mercury thermometer for chamber monitoring is not practical because it violates animal health and well-being requirements and lab safety regulations.

Oxygen detection and calibration was done by pulling the voltage from a Teledyne R17A oxygen sensor using an analog-to-digital converter (ADS1115). The Teledyne R17A has a functional range of 0–100% oxygen at 25°C with a resolution of 0.1% oxygen (Model R-17A, 2022). The voltage being recorded by the ADS1115 gets converted using the oxygen calibration data. Oxygen calibration was achieved by connecting a Teledyne R17A oxygen sensor to a hose fitting attached to a flow regulator to generate oxygen concentrations of 30, 15, 10, and 5% oxygen to develop a standard curve. Additionally, if a flow regulator is not available, the oxygen sensor can also be calibrated with premix tanks (10 & 5%) and room air (20.9%), although room oxygen is unreliable. To generate a standard curve using oxygen tanks, we attach one of the oxygen sensors to a manifold and tubing connected to tanks with different gas mixtures and expose the sensor to a given O_2_ concentration for 10 min (Teledyne recommendation). The oxygen calibration script will take a series of samples every 10 s and then average the voltages to get a voltage value for the concentration being exposed. Repeat the process for at least three O_2_ concentrations then create a standard curve to be used in percentage calculations. Once we established the calibration curve, the oxygen sensor’s output was measured and calculated based on the standard curve to determine the actual oxygen concentration (Figure 1D).

**FIGURE 2:**
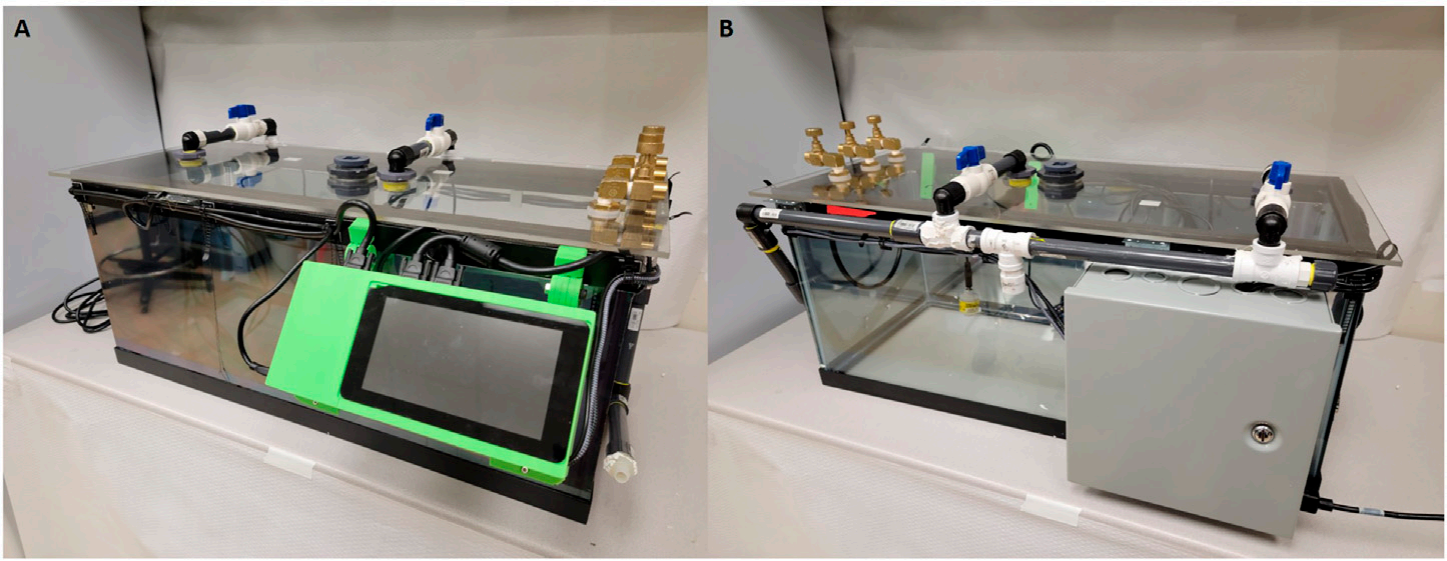
Images of current cage design including updated sensors and revised power supply. **(A)** Front view of the chamber with a 7 inch Raspberry Pi touch screen and visible airflow systems. **(B)** Rear view of the chamber with power supply container and airflow input tubes visible.

Light calibration is essential for quantifying light/dark cycle and understanding changes in physiology related to diurnal cycling. Obtaining the light by measuring lux allows us to determine if precise day/night cycles are adhered to within our animal care facility, again as a way to control the chamber environment and reduce treatment variability. To detect light the system utilizes adafruit BH1750 sensor boards (Siepert, 2021). The sensor board has a range from 0.11 lux minimum to 100,000 lux minimum with a noise reduction of 50–60 Hz. The sensor variation is ±20% with minimal infrared interference (Siepert, 2021). Additionally, the chamber walls were treated with a one-way mirror reflective coating to help minimize animals’ perception of handlers during the hypoxia exposures (Figure 2). Any variant of one way mirror film can be used to treat the chamber walls.

### Data Acquisition

Each sensor has its own function written in a master *Python* program which specifies the data recording resolution and interval that each sensor measures. These values are assigned to variables and it is easy to calculate calibration curves and obtain precise, calibrated measurements for each experimental condition measured by the on-board sensors. Our data acquisition cycle for the chamber is episodic every (10 min), using a function that saves the start and end times from the Raspberry Pi system clock and then calculates the time required to collect all the data and average over the 10 min epoch. The sampling rate can be readily adjusted within the Python program by changing the sampling interval. The data is acquired and stored throughout each 10 min acquisition period. All data is saved as comma-separated ASCII text files (.CSV) with values for raw, unprocessed data, and processed data with specific measurements. Storing data in a text file allows a great deal of flexibility since data analysis can be performed using *Python*, *R, MATLAB* or other analysis software. The caveat is, of course, that text files take up more disk space than binary files. It would be easy to add a compression function to make the files smaller but that is not currently included in our code as we use gzip or bzip2 to batch compress data files after checking them. The sensors are mounted on custom PCB boards (Central, Top, and External), which connect all the sensors back to the RaspberryPi via VGA cables (Figures 1B, C) which provide excellent shielding in a compact form factor.

The chamber is designed with a breakout PCB board to convert the 40 pinouts to four VGA connectors allowing for four different sensor PCB breakout boards to be attached depending on researcher needs.

### Results and Analysis

**FIGURE 3:**
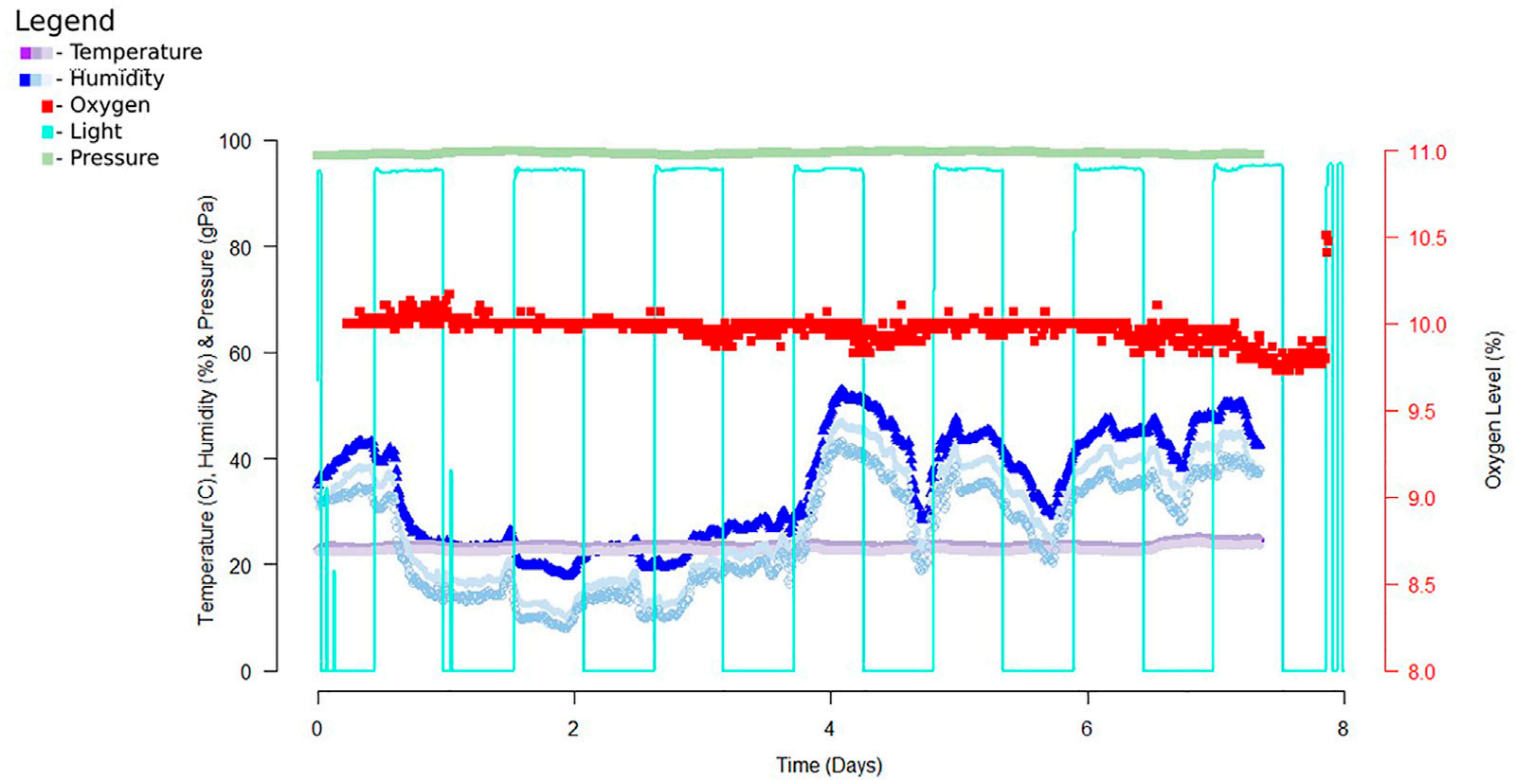
Example data from a 7-day exposure of the chamber. Data was recorded using a Python script then analyzed with R studio. The data recorded above reports data collected from early experiments utilizing DHT22, ADS1115 and Teledyne oxygen sensor, TSL2591, and MPL3115A2. For this dataset we recorded data every 10 minutes for the entire 7 day exposure time. Since completion of this sampling we replaced these sensors with sensors of higher accuracy and precision.

Chamber monitoring allows us to record and monitor the environmental conditions within the chamber during extended hypoxia exposure, providing the ability to make real-time adjustments to the chamber’s environment. During preliminary tests of the chamber, graphical time-series plots were generated to compare temperature, humidity, oxygen levels (Figure 3). *Python* and *R* were used to analyze and summarize chamber temperature and oxygen level data and identify changes in environmental conditions. We utilized the *Raspbian* operating system (based on *Debian GNU/Linux*), which provides remote access to the chamber software/hardware using secure shell (*ssh*). With the *Python* script collating reported values in a text file, custom analysis can be done using a variety of programs, including *Python, R, Excel*, *LibreOffice*, *GraphPad Prism,* etc. according to the user’s preferences.

In our analysis, we focused on identifying time points where oxygen deviated from our tolerance of 10 ± 0.5% O_2_. Deviations from our target oxygen levels were compared to other environmental variables to determine if a change in the environment was altering oxygen levels. In order to get accurate readings, oxygen concentration was determined by two-point calibration of the sensor using a vendor-supplied tank with a custom oxygen mix. The data collected provides a post-exposure view of the environmental condition of the chamber. As shown in Figure 2, the chamber allows for a controlled environment that can house multiple cages, for consistent exposure across several animals simultaneously, allowing us to reduce variability between exposure groups.

### Control of Environment Variables

Environmental regulation is important in any experiment and being able to record multiple environmental variables can facilitate reproducibility and reliability of data. During early experiments, we found that a consistent temperature and oxygen levels led to higher survival rates in our experimental animals. This observation was supported by previous studies showing that a cold environment can lead to increased stress and affect the immune system (Meyer et al., 2007; Hankenson et al., 2018). During our hypoxia exposures, the animals have already undergone an immune challenge, so the addition of temperature stress can increase the stress on experimental animals in a given protocol. Due to this, we wished to monitor and control as chamber environment variables as possible. Environmental stress during development has been attributed to psychological stress in rodents as well, so minimizing this stress is critical in most studies (Takuma et al., 2011). The animals’ environment has also been shown to affect their immune response when exposed to an inflammatory challenge (e.g. a “two hit” protocol or more complicated exposure paradigm); thus it is important to provide a controlled environment for experiments in which animals are exposed to stressors (Mueller et al., 2018).

Finally, our goal has been to provide an affordable chamber with supporting hardware and software for high-resolution data acquisition and reproducible results for experiments requiring O_2_/CO_2_/N_2_ gas and monitoring of other environmental variables. By monitoring the environment for experimental animals, we provide a platform for more reproducible and cost-effective experiments. Alternatives that are currently used include plethysmography chambers, both head-out and whole-body, for monitoring breathing during hypoxia/hyperoxia exposure. Our open-source system provides a more flexible foundation for rigor, transparency, and reproducibility. Commercial exposure systems can be very expensive and the investigator is often limited by their budget for multiple animal exposures. Our system lowers the cost of multiple animal exposures significantly while providing high-resolution data from the suite of sensors we have specified. We are not suggesting that our system replaces more sophisticated commercial exposure systems but, instead, provides a cost-effective alternative. Commercial plethysmography systems can provide a more extensive array of sensors and data acquisition (Ortega-Porcayo et al., 2014), and we will continue to develop and expand upon our open-source system. Finally, the ability of an open-source system allows for high customizability of the apparatus for each researcher’s needs. Collaboration between labs utilizing this system can help us improve the chamber and control system while also allowing for new laboratories to develop their own custom systems (Supplementary Table S1).

## Future Directions

The hypoxia chamber we describe has the ability to monitor and acquire data at high resolution and provide a way to standardize exposure protocols inexpensively by virtue of FLOS hardware and software. The chamber hardware and software are in constant development and we look forward to feedback for the development of additional features. An additional feature that would enhance the flexibility of our system would be to add a feedback controlled humidifier to control humidity in the chamber. A movable camera would also provide enhanced monitoring of experimental animals and a way to assess behavior and health of the animals. We also could add sound frequency and pressure measurements within the chamber. The addition of a white-noise sound suppression system to reduce the effect of environmental noise within the chamber and could mask environmental noise that might be a stressor. Including more sophisticated temperature control may be useful for some investigators and that should be possible with minimal changes to the current control circuitry and software. Finally, we intend to develop a graphical user interface (GUI) front-end to the control program to make it easier for non-technical users to use. While the chamber has several improvements that are in progress, the current system allows investigators with limited budgets to develop flexible exposure protocols and easily acquire environmental data. Even a minimal monitoring set-up will allow for a more uniform exposure system across typical hypoxia-based studies (Vannucci and Vannucci, 2005; Gunn and Bennet, 2009; Lacaille et al., 2019).

## Conclusion

We have described a low-cost hypoxia exposure chamber that allows for high-resolution data monitoring and reporting of environmental status. Our goal in developing this chamber and associated control systems, was to provide a highly reproducible, FLOS system to facilitate low-cost physiology experiments. Use of FLOS software and hardware allows for improvements, customization, and tailoring of the chamber and software for specific experimental conditions as needed in each laboratory. We hope that, by providing this system, we can stimulate collaboration and continuous development of the chamber hardware and software. We designed the system to have a usable, minimum set of sensors acquiring temperature, humidity, light, pressure, tVOC and oxygen levels. In the future, we hope to include real-time movement monitoring to make the system more attractive to a wider range of investigators as these factors have a significant impact on experimental animals (Mueller et al., 2018). We will continue to develop this system and seek input from other investigators to maximize the system’s versatility and usefulness for open, reproducible science. Our hope is that other investigators will benefit from our work and contribute to our on-going efforts to improve and expand upon the chamber hardware and software.

## Data Availability

The datasets presented in this study can be found in online repositories. The names of the repository/repositories and accession number(s) can be found below: https://github.com/drcgw/hypox-chamber.
