# Supplementary material for: An Inexpensive Open-Source Chamber for Controlled Hypoxia/Hyperoxia Exposure
Source: Front Physiol. 2022 Jul 12;13:891005. doi: 10.3389/fphys.2022.891005 (PMC9315218; doi:10.3389/fphys.2022.891005)
Supplement: Supplementary file 1 [file DataSheet1.PDF]

Instructions for build and assembly of Hypoxia Chamber

Version 1.003, Edited: 5/29/2022, Created: 2/22/2022

Tyler C Hillman[1], Ryan Idnani, and Christopher G Wilson[1,2]

1. Lawrence D. Longo, MD Center for Perinatal Biology

2. Department of Pediatrics, Loma Linda University Medical Center

Loma Linda University, School of Medicine

Loma Linda, CA 92350

Corresponding Author:

Christopher G. Wilson, PhD

11223 Campus St

Loma Linda CA. 92350

Desk: 909-651-5895

Email: cgwilson@llu.edu

Supplies and equipment:

**Item Name**	**Description**	**Vendor**	**PN**	**Cost**	**Quantity**	**Link**
Cage Structure						
Glass Tank	Aqueon Glass Aquarium Tank - 20 Gallon	PetCo		$ 49.99	1	20Gal-Tank
Glass Tinting	One-way window film	Amazon		$ 11.99	1	Film
Acrylic Sheet	Chamber top - 1/4 x 31.5 x 14.25 in	ACME Plastics		$ 45.00	1	Acrylic
Junction Box	Steel Junction Box, 254 x 254x 101.6 mm	Newark	40T7867	$ 31.60	1	Junction box
Cable Management	3.35 inch black cable straps	Amazon	B0881FW8TQ	$ 9.99	Variable	Straps
Airflow						
Bulkhead	1/2 in female PVC bulkhead fitting	Amazon		$ 12.49	2	Bulkhead-0.5in
PVC Tubing	1/2 in FPT T joint	HomeDepot		$ 1 - 3	3	
	1/2 in MPT Valve	HomeDepot		$ 1 - 3	2	
	1/2 in MPT Adapters (1/4, 5 and 10 in)	HomeDepot		$ 1 - 3	8	
	1/2 in MPT/FPT Elbow	HomeDepot		$ 1 - 3	6	
Brass Tubing	1/4 in Bulkhead	Amazon		$ 12.89	2	Bulkhead-0.25in
	1/4 in R-angle Elbow	Amazon		$ 14.59	2	Brass-Elbow
	1/4 in Control Valve	Amazon		$ 14.99	2	Control Valve
Electrical						
Sensor PCB*	Custom PCB boards for sensor attachment	OSH Park		$ 102.50	5	
Raspberry Pi**	Raspberry Pi 4B, 2 GB RAM	Adafruit	4292	$ 45.00	1	RPi
PiCamera	Raspberry Pi Camera 2	Adafruit	3099	$ 29.95	1	PiCamera

Item Name	Description	Vendor	PN	Cost	Quantity	Link
Power Supply	Adjustable DC Powersupply 110V - 220V, Output: 0 - 24 V, 20A 480 W	Amazon	B08GFSVHLS	$ 35.99	1	Power
Electrical						
MCP9808	High accuracy I2C Temperature Sensor	Adafruit	1782	$ 4.95	3	MCP9808
BH1750	Light Sensor I2C	Adafruit	4681	$ 4.50	2	BH1750
BME280	Temperature, Humidity & Pressure sensor I2C	Adafruit	2652	$ 14.95	2	BME280
CCS811	VOC and eCO2 Sensor	Adafruit	3566	$ 19.95	2	CCS811
ADS1115	4 Channel 16 bit Analog to Digital Converter	Adafruit	1085	$ 14.95	1	ADS1115
Voltage Regulator	3.3V 800 mA Voltage regulator	Adafruit	2165	$ 1.25	5	LD1117
0.1 uF Capacitor	0.1 uF capacitor, max of 50 V	Adafruit	753	$ 1.95	5	0.1uF Capacitors
10 uF Capacitor	10 uF capacitor, max 50 V low-frequency	Adafruit	2195	$ 1.95	5	10 uF Capacitors
Power Jack	Gravitech DC Power Connector 2.1mm x 5.5mm	Mouser	992-CON-SOCJ-2155	$ 1.00	5	Power Connector
D-Sub 15 Female	D-Sub High Density DBHD15, Female	Amazon	CNR15HDM-F-10PACK	$ 12.88	8	D-sub 15
VGA Cables	UGREEN VGA, 3 meter, male to male coaxial	Amazon	B00OZL3HLO	$ 7.99	4	3ft VGA
USB Cables	Micro-USB Cable, R-angle joint	Amazon	B09C5P7YFN	$ 9.99	1	microUSB
USB Cables	USB-C Cable, R-angle joint	Amazon	B09CG9LZSR	$ 9.99	1	USB C
DC Power Cable	DC Power Pigtail, 3ft, Male 5.5mm x 2.1 mm plug	Amazon	B08PYWN3T7	$ 9.99	4	Power Cable
DC Cable Extender	6 ft DC Extension Cord, male to female	Amazon	B074WJZNZD	$ 13.99	3	Power Extension
Hardware						
M6 Bolts	M6x30 Bolts	Grainger	26LG83	$ 8.72	2	M6X30

Item Name	Description	Vendor	PN	Cost	Quantity	Link
	M6 Washers<br>M6 Nuts		26WC34<br>22YK33	$ 2.01<br>$ 2.31	4<br>2	M6 Washer<br>M6 Nut
M3 Screws, Washers & Nuts	M3x10<br>M3x12<br>M3x18<br>M3x20<br>M3 Insulated Gasket<br>M3 Nut	Granger<br><br><br><br>Amazon<br>Granger	22TY49<br>22TZ12<br>22TZ14<br>22UC27<br><br>22UK74	$ 4.09<br>$ 4.09<br>$ 4.40<br>$ 4.99<br>$ 7.99<br>$ 2.68	6<br>1<br>2<br>3<br>8<br>6	M3X10<br>M3X12<br>M3X18<br>M3X20<br>M3 Gaskets<br>M3 Nut
M2.5 Screws, Washers & Nuts	M2.5x6<br>M2.5x8<br>M2.5x10<br>M2.5x12<br>M2.5 Washer<br>M2.5 Nuts	Grainer	22TX95<br>22TX96<br>22TY34<br>22TY35<br>22UE65<br>6CA67	$ 3.47<br>$ 3.81<br>$ 3.19<br>$ 2.27<br>$ 2.07<br>$ 2.32	2<br>4<br>4<br>10<br>12<br>18	M2.5X6<br>M2.5X8<br>M2.5X10<br>M2.5X12<br>M2.5 Washer<br>M2.5 Nut
4 # Screws & Standoffs	4#-40*5+7 standoff screws<br>4# Screws	Amazon		$ 16.98	4<br>2	Standoff

Chamber Preparations

Step One: Preparation of the lid

- Once the acrylic has arrived, measurements and marking should be done to identify where on the lid you are going to make holes for various aspects of the chamber.

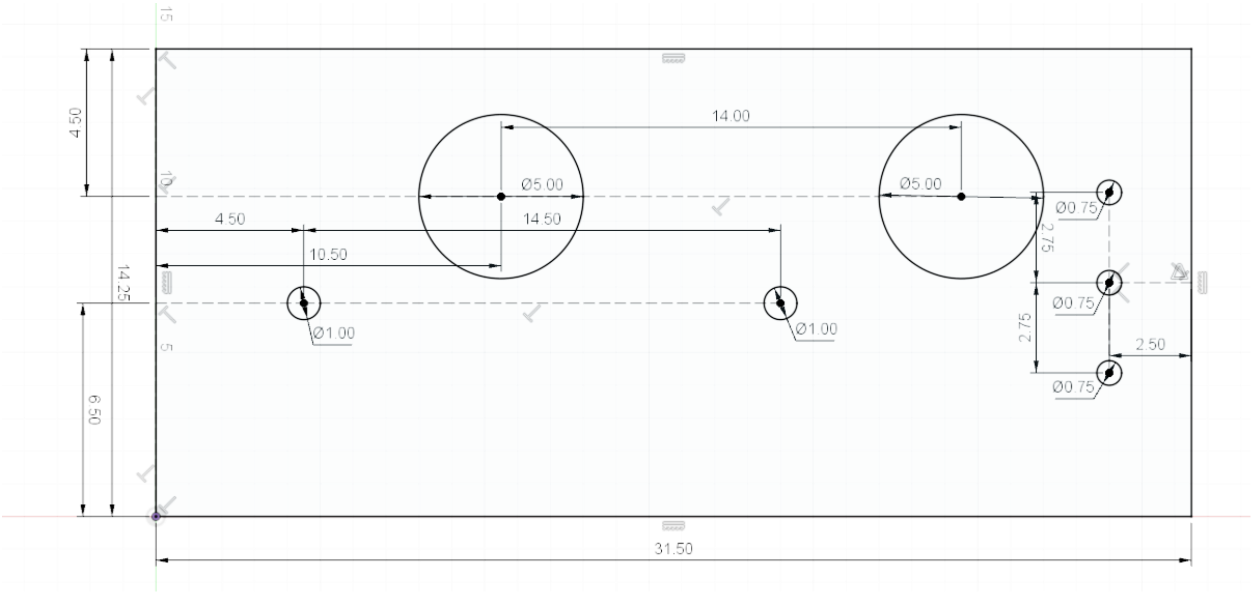

- Once centers have been measured, apply masking tape to the surface of the acrylic and cover with a light layer of mineral oil.
- Using an electric drill and hole drill bit, gently drill holes into the plexiglass. When doing this process, consistent speed and a well-oiled bit is important. Do not apply too much pressure as you can fracture the acrylic.
- After each hole is drilled, take a general quick set epoxy and add a layer on the inside and a 5 mm ring on the top and bottom; this will help seal any microfracture that may have been created.
- Depending on where you want the connections, put together the PVC air inflow. This is customizable and can be as simple as connecting the two with the input into the center or making it more elaborate to feed in on the side of the chamber.
- Slide the bulkheads into place after the rest of the plumbing is attached. Secure with the lower bult of the bulkhead and insure it is tight. Do not over tighten.
- Add the brass bulkheads. Use the silicone O-rings to help create a stronger seal.
- Optional Arm Holes:
    - If you would like to have access to the chamber through arm holes, the large PVC DMV Spigot w/ plug adapter fitting can be installed to give lid access.
    - When drilling the hole in the lid of the acrylic, ensure that the hole drill and acrylic are always lubricated, which can be accomplished with water or mineral oil.
    - The size of the whole is dependent on spigot size, we utilized an internal diameter of 4 inches, and a hole dril of 4.5 inches.

    - Ensure that when drilling even weight is applied and the drill is in an upright position, 90 degrees from the acrylic.
    - Do not apply too much pressure and try to avoid the drill catching.
    - Once the hole is created apply a layer of epoxy to the edge to seal the surface.
    - Before installing the spigot, a bead of 100% silicone needs to be put on the lip of the spigot then carefully slide into the acrylic sheet.
    - Once on, add additional silicone and smooth w/ finger to insure a good seal
    - Let sit for 24 hours before moving on.
    - Flip over and add another bead of silicone around the bottom to create a seal, allow to set for 24 hours.
- Once all of the through attachments have been completed, add ⅕ inch thick weather seal stripping to the edge to create a seal between the lid and chamber bottom.
- This is the completion of the basic chamber aspect. The next section will go into the addition and creation of the electrical components.

Step Two: Preparation of the chamber bottom

- The only addition that needs to be added to the chamber base is one-way mirror film. Measure the length and height of the glass pain on the side of the chamber.
- On the one-way mirror film, measure the distance of the glass with an additional 1 inch to the measurement (or 10 mm if in metric; this distance is not critical and will be trimmed in a later step).
- Clean glass with soapy water and 100% ethanol. Once cleaned, apply adhesive spray and follow the one-way mirror films instructions for application. Clean the edges so they are even with the chamber.
- Only do this for 3 sides of the chamber if you are going to add the electronic components. Installation of the power supply box will be covered in a later step.

Step Three: Preparation of the PBC boards

- All of our sensors were ordered from Adafruit and require assembly.
- We recommend using a fine solder tip on your soldering iron and a solder that has a rosin core (between 0.5 mm and 0.8 mm diameter).
- Using 90-degree pins solder the boards and pins together
- Once all of the purchased components have been assembled, they can be soldered onto the custom PCB boards we created for this project.
    - All the boards are available on GitHub (https://github.com/drcgw/hypox-chamber.git).
    - We ordered through OSH Parks, a custom PCR prototyping company (https://oshpark.com/).
- Each board has the name of the sensors to be placed in each pin out slot. Make sure that the pinouts on your board match those on the sensor breakout and then solder the boards on the custom PCB boards.

- We have provided the designs for 5 different boards, WE DO NOT USE ALL FIVE. These boards are designed to give you the option to customize based on need.
- Our chamber uses VGA-HAT, External, Top and Central which houses a series of sensors on each.
- However, if a simpler chamber is needed the MCP only board can be used.
- The HAT-VGA board allows for conversion of the 40 pinout to VGA ports and is recommended for use with all sensor board breakouts.

Step Four: Raspberry Pi, Pi Screen and Raspberry Pi holder installation

Step 4.1 (Adapter installation):

- The first installation step is the addition of the PiCamera to HDMI adapter.
- On the smaller 3D printed holder which can be downloaded from (https://github.com/drcgw/hypox-chamber.git). Recommended print settings are: 25% infill, 0.1 mm layers, PETG filament (Monoprice), with a print speed at 75%. When splicing, print the files lengthwise and avoid using support materials.
- First create four stacks of two M1 standoffs. Place the converter with the HDMI connect oriented down. Secure the ribbon cables and converter with the small M1 screws.
- See above image for location references.

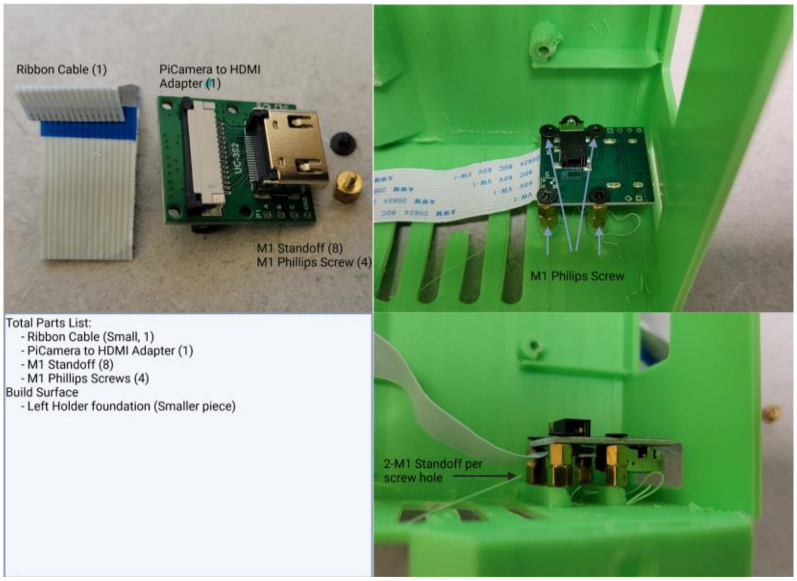

Step 4.2 (Screen fan installation):

- The next piece to be installed will be installed in the larger of the two 3D printed screen holders.
- Flip the holder so the screen side is facing up, the fan will be mounted on this side with the air flow being directed towards the screen.
- Slide four M2.5x10 screws into each of the four screw holes and secure them with M2.5 nuts and washers on the screen back side of the holder.

- Ensure the power cable is run towards the screen back side of the holder.

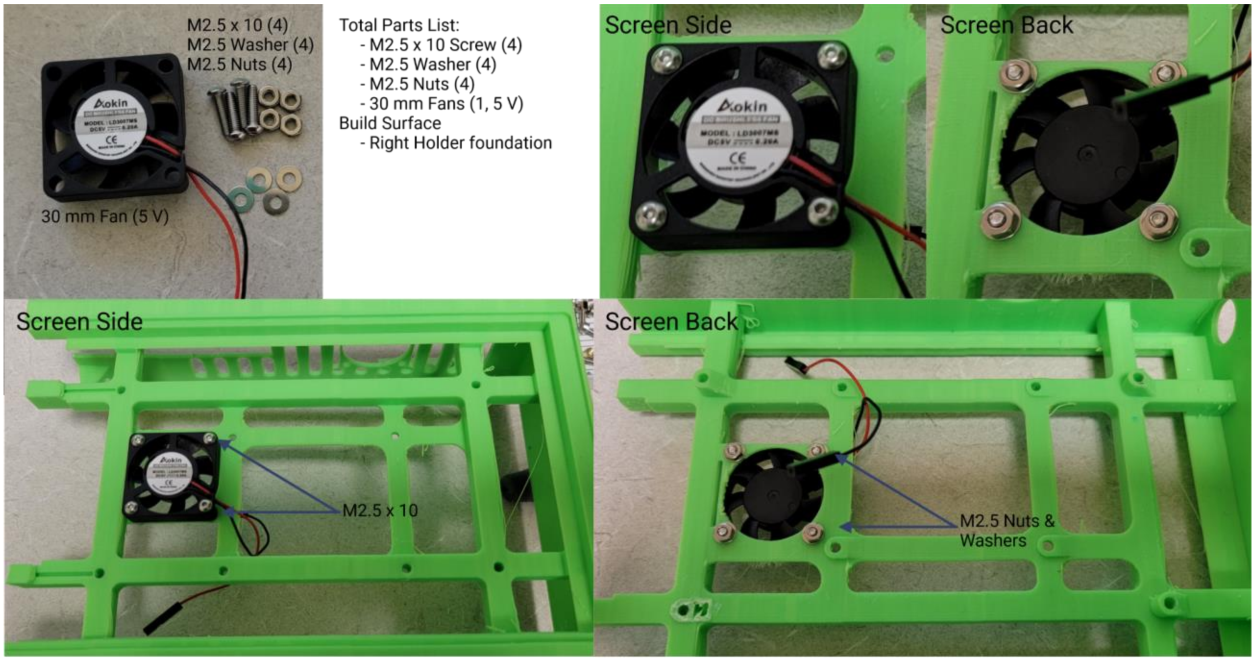

Step 4.3 (Screen Installation):

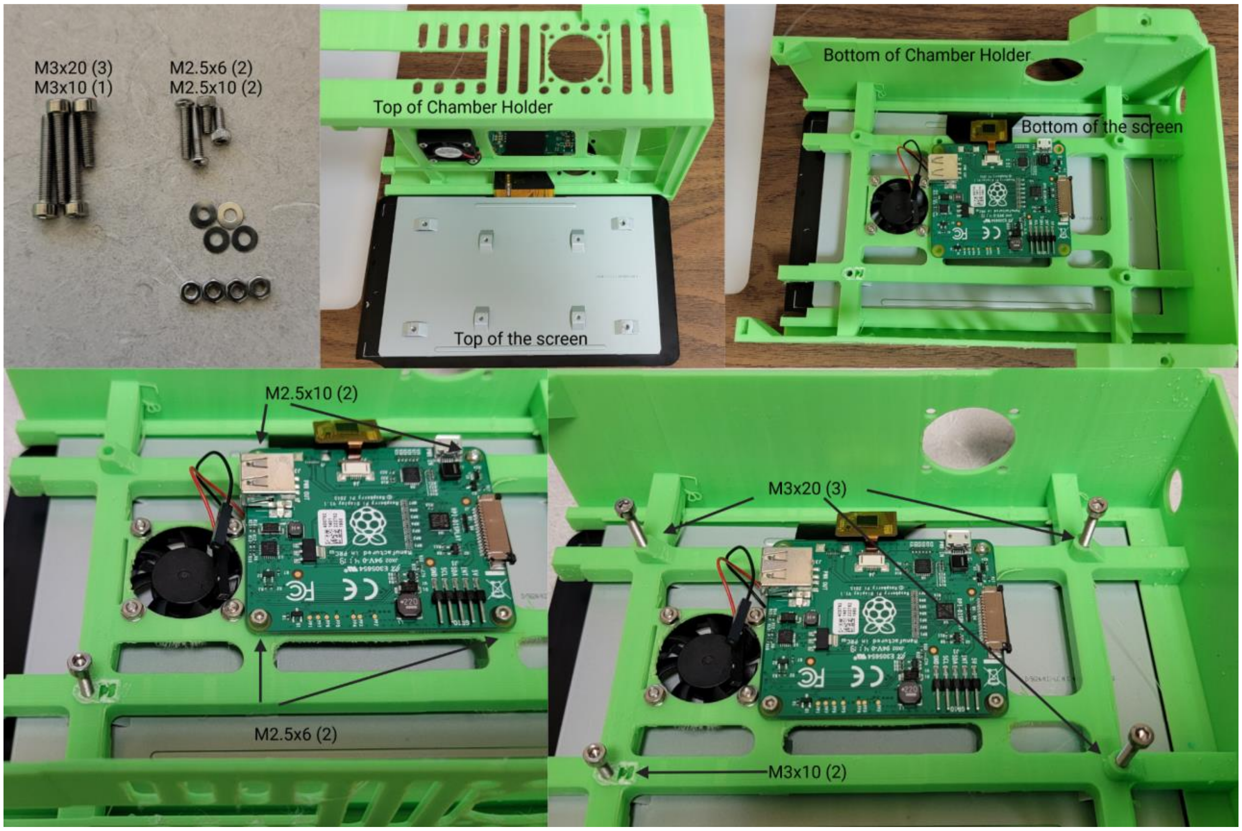

- The bottom of the screen will have a ribbon cable attached to a control board. The top of the 3D printed holder has large gaps for the VGA cables to ensure that when installing the screen, the bottom of the screen is at the bottom of the holder (Top central image).
- Slide the control board through the gap between the holder side and support bar.
- Once the screen is in place, lay the holder over so it is sitting on top of the screen and the anchor holes align.
- Place the screen control board onto the support beams and align the screw holes. Using two M2.5x6 screws slide them through the control board and holder supports, secure using two M2.5 nuts and washers (Please note these go on the two holes closest to the screen holder top).
- Once secured, return it to the laid down orientation and slide two M2.5x10 screws into the other two control boards and through the supports, secure using two more washers and M2.5 nuts.
- The screen is secured using three M3x20 screws on the holes with raisers and a M3x10 screw on the one without a raiser.
- When screwing in, make sure that the screws go all the way through the supports before applying gentle pressure on the screen allowing the screen to be attached to the supports. DO NOT OVER TIGHTEN THE SCREWS. If you do, you will forcibly pull the back of the screen off the screen.

Step 4.4 (Connection of two screen holder pieces)

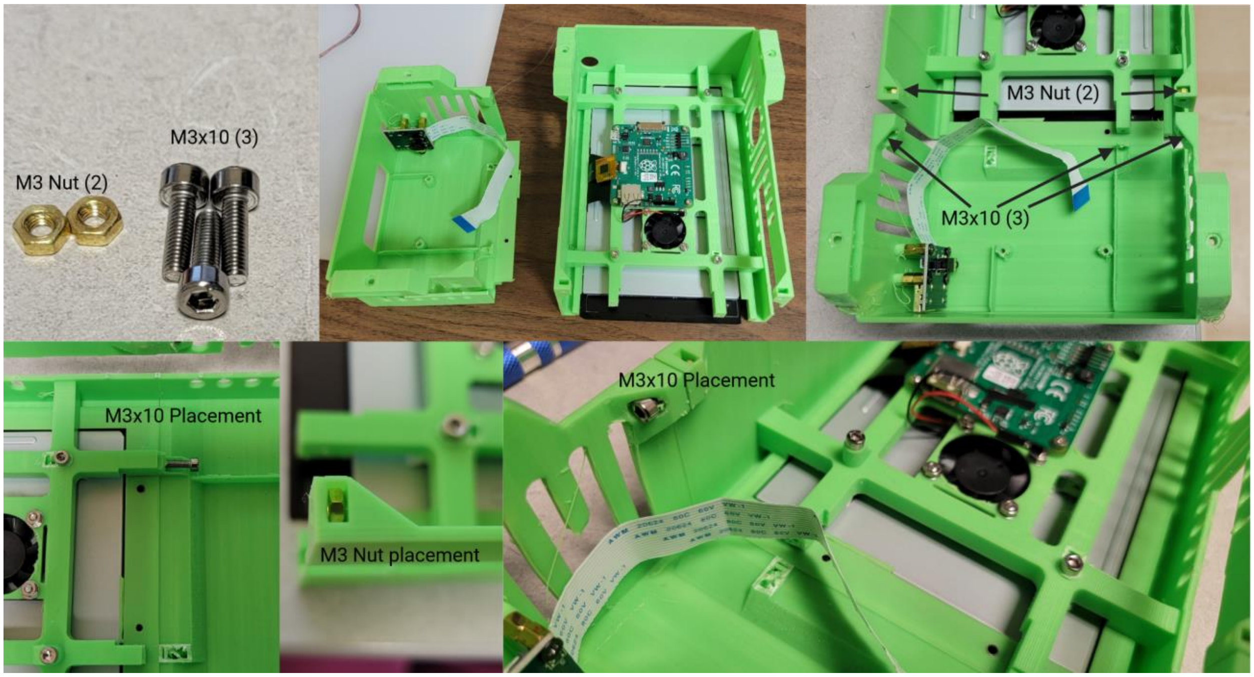

- The two screen holder components slide together as shown in the far-right top image.
- Slide two M3 nuts into the holes shown in the top far right image.
- Using three M3x10 screws, connect the two pieces as shown above.

- Tighten to secure.

Step 4.5 (Fan installation)

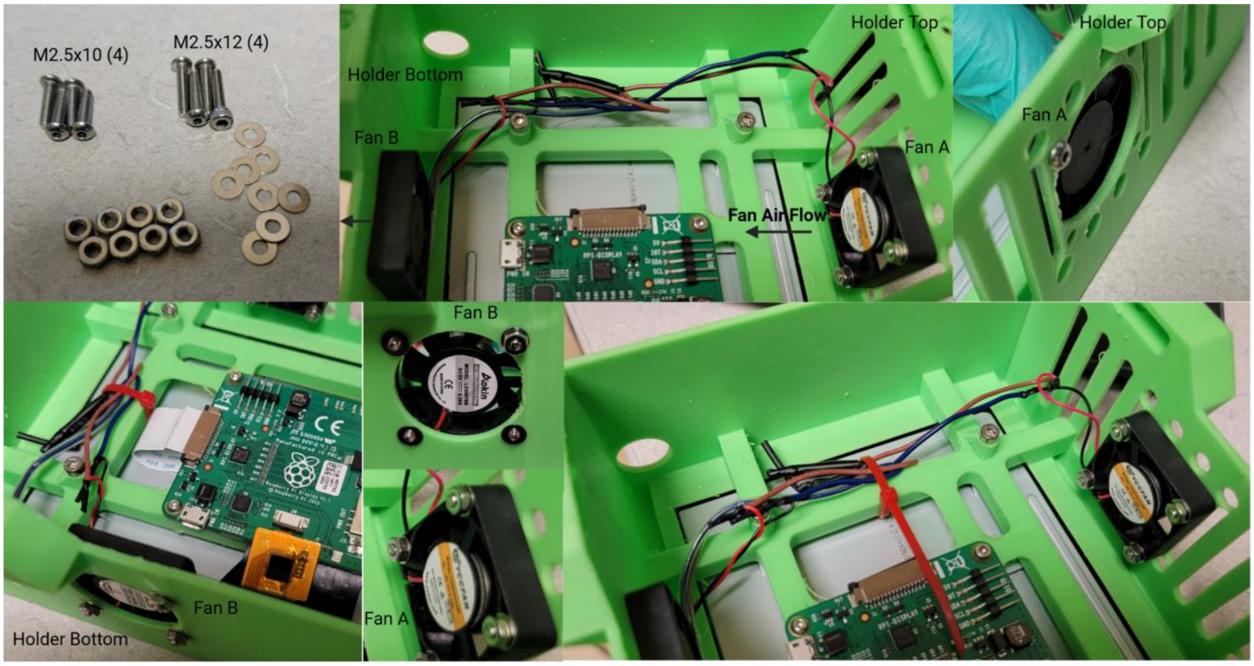

- Two additional fans are used to aid with air flow throughout the screen holder.
- Before beginning installation, connect the two fans by stripping the wires and connecting them using solder and heat shrink tubing. Connect them to some wire to extend the length by 200 mm, next attach Dupont connectors to the end of the wire not connected to the fans.
- Once completed, attach the fans at the fan ports above and below the screen control board.
- Attach the fans in the same direction.
- The fans can be attached with four M2.5x12 screws, washers, and nuts; or one can be attached with M2.5x12 screws and the other attached with M2.5x10 screws.
    - To use the M2.5x10 screws on the fan, fan B can have the screws slightly embedded into the fan folder and the nuts secured on the outside of the holder.
    - If using two sets of M2.5x12 screws, then orientation does not matter.
    - In the above diagrams, Fan A is secured using M2.5x12 screws with the screw heads being on the outside of the holder and washers/nuts securing the fans on the inside of the holder.
    - Fan B is secured by embedding the head of the M2.5x10 screws into the fan casing then securing the fans using washers and M2.5 nuts on the outside of the holder.
- Once fans are secured, secure the wiring by attaching it to the supports with a zip tie.

- Additionally, during this stage, connect the ribbon cables into the control board and slide it under the control board till it comes out on the other side of the screen control board. This orientation will be needed for future steps.

Step 4.6 (Raspberry Pi installation)

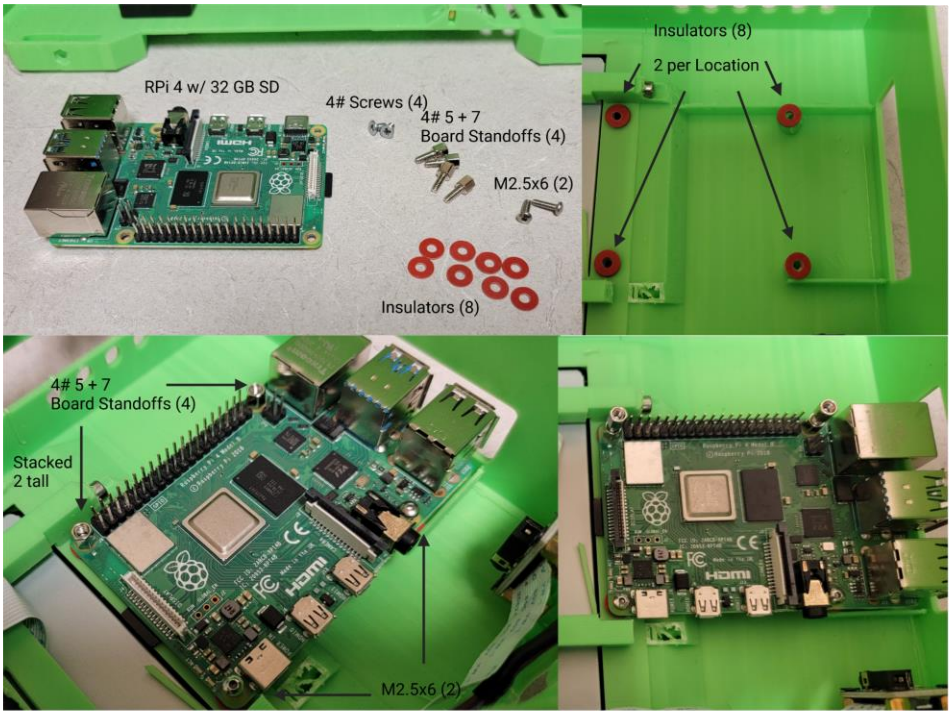

- The Raspberry Pi needs to be elevated to allow for the microSD card to fit. In order to accomplish this, 8 insulators will be used over each of the RPi attachment stands.
- Onces the insulators are in place gently place the RPi onto the holder and attach the bottom two using M2.5x6 screws. You will need to secure them tightly before proceeding.
- Using a socket wrench secure the top of the RPi using 4# standoff screws. Once secured, connect a second onto the standoffs just installed. This is present to support the VGA-HAT.

Step 4.7 (Ribbon cable attachment)

- Two ribbon cables need to be connected to the Raspberry Pi, the display, and the camera.
- First, connect the display ribbon cable onto the connector just above the microSD card as seen in the top right image. The metal contacts on the display should be pointed towards the USB ports and away from the microSD card.
- Second, connect the camera ribbon cable to the port between the microHDMI and auxiliary ports. For this, ensure that the metal contacts are facing away from the USB ports.

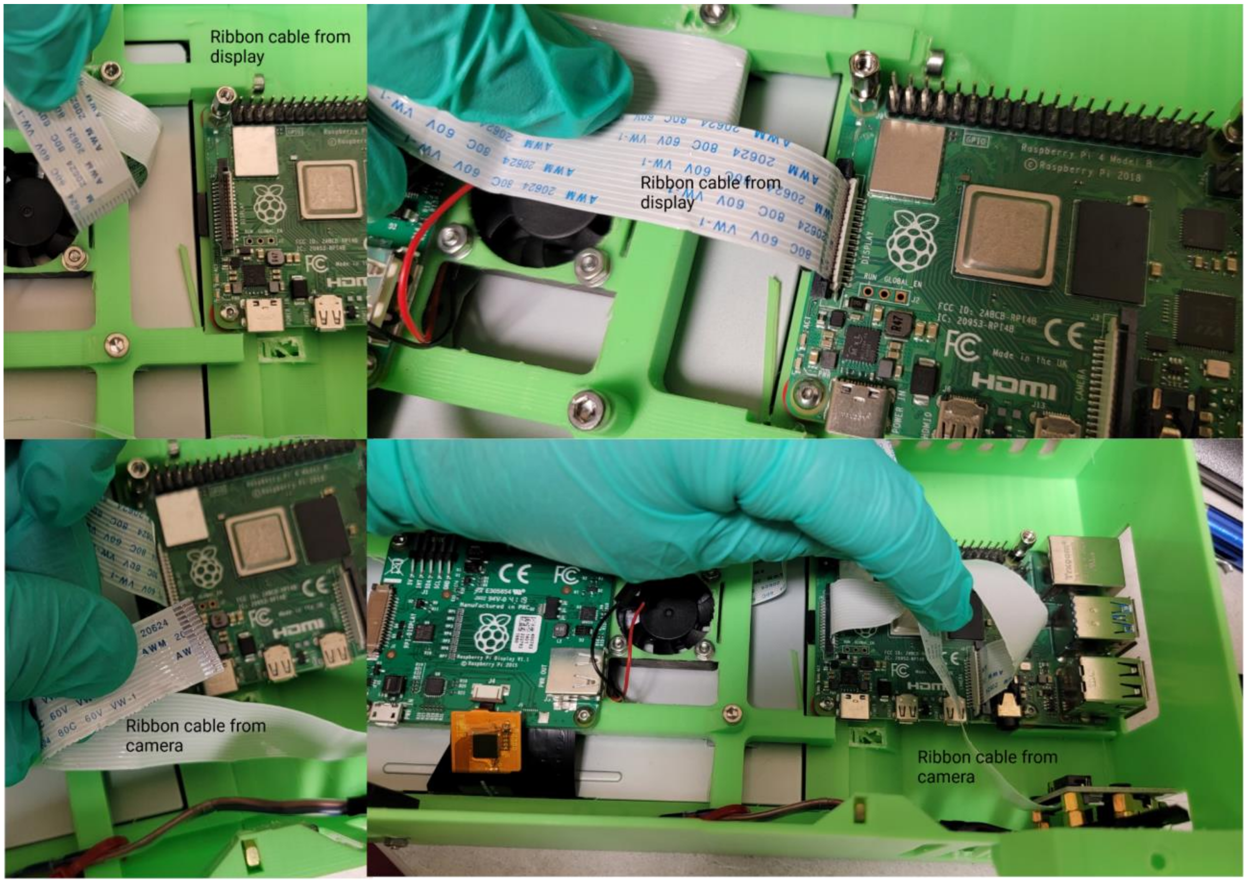

Step 4.8 (VGA-HAT installation)

- In order to prepare the VGA-HAT, a fan needs to be installed.
- Ensure that the fan is pointed towards the 2x20 female adapter, which will be directed towards the Raspberry Pi.
- Embed the screw heads from the M2.5x10 screws into the fan and then slide this through the PCB board. Once through, secure using a washer and nut to the screws.
- Place the 2x20 connector onto the

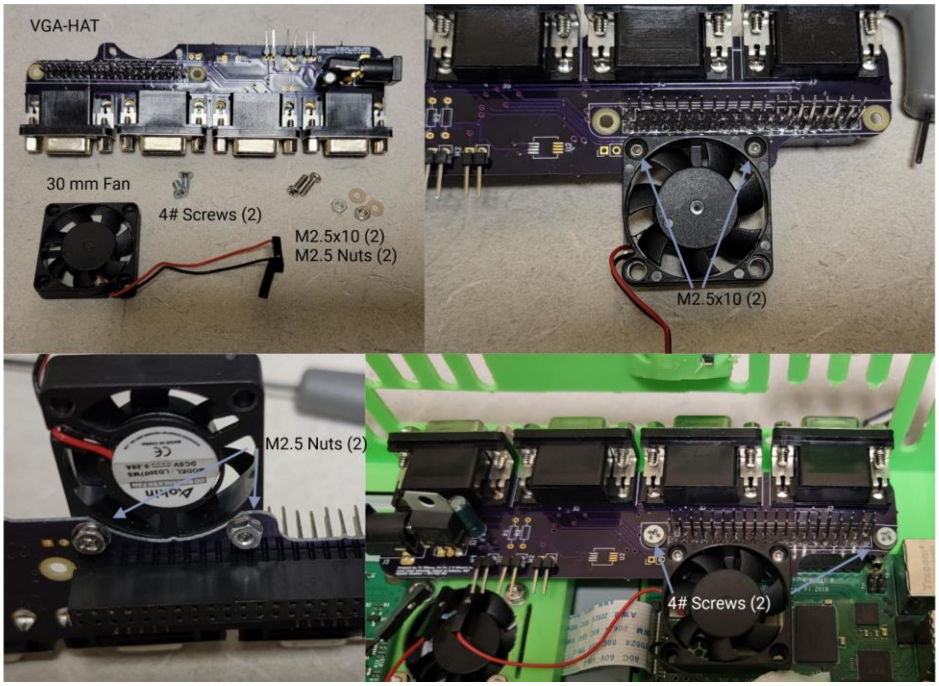

RPi's GPIO pinout. When setting this up, make sure all the pins align and the VGA connectors are directed out towards the screen holder's top.
- Hold it in place with two 4# screws.

Step 4.9 (Electric assembly)

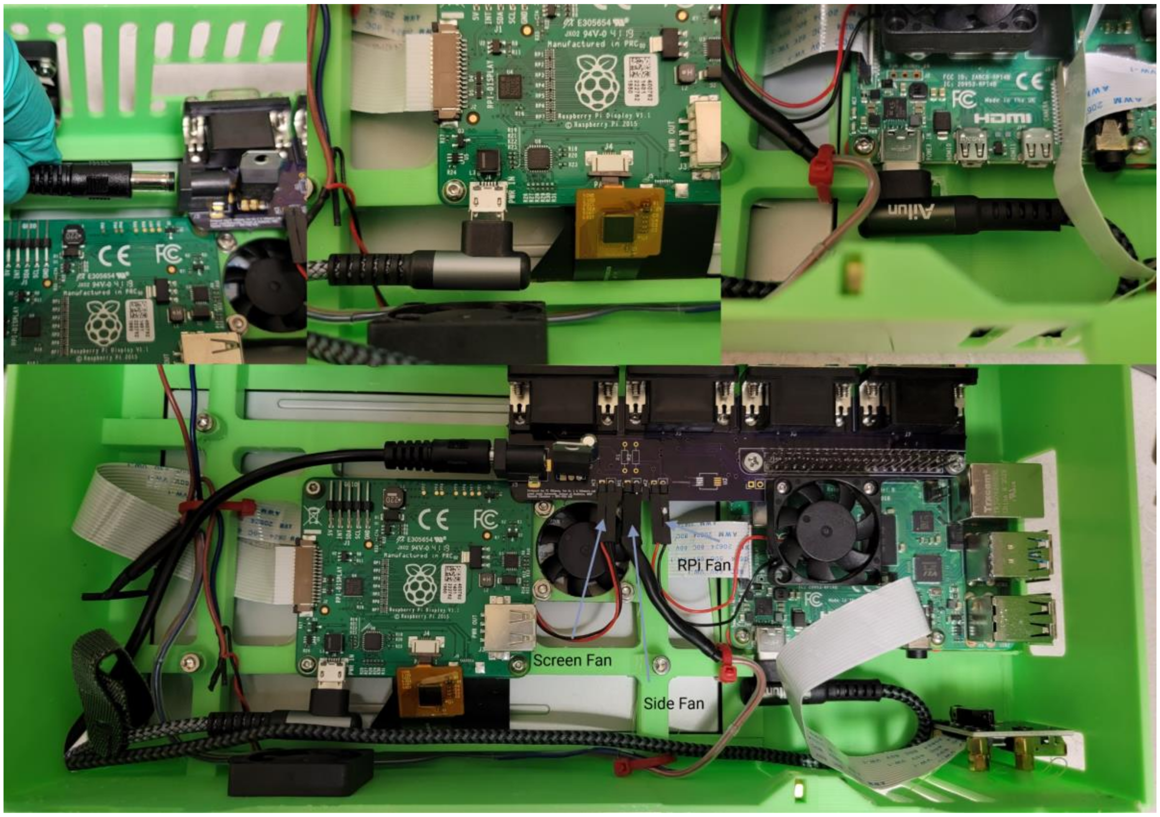

- There are six different power cables that need to be attached.
    - IF USING AN ADJUSTABLE POWER SUPPLY, CONNECT THE POWER SUPPLY TO POWER AND SET FOR 5.0 V BEFORE CONNECTING ANY POWER. SHUTDOWN THE POWER BEFORE MOVING FORWARD.
- First, connect the power supply cable into the VGA-HAT power connector.
- Second, connect the microUSB cable to the power connector on the screen control board.
- Third, connect the USB-C cable to the power connector on the raspberry pi (FOR RPi 4 **ONLY**. If using another RPi version, please determine power supply needs and use the correct cabling. Failure to do so could lead to destruction of the RPi).
- Fourth, connect the fan power cables to the three power supply pins on the VGA-HAT.
    - The screen fan should be attached to the first set of pins with the power first, followed by ground.
    - The side fans should be connected to the second set of pins, with the first being connected to power and second to the ground.

- Finally, the RPi fan should be connected to the third set of pins with power first and ground second.

Step 4.10 (Chamber attachment)

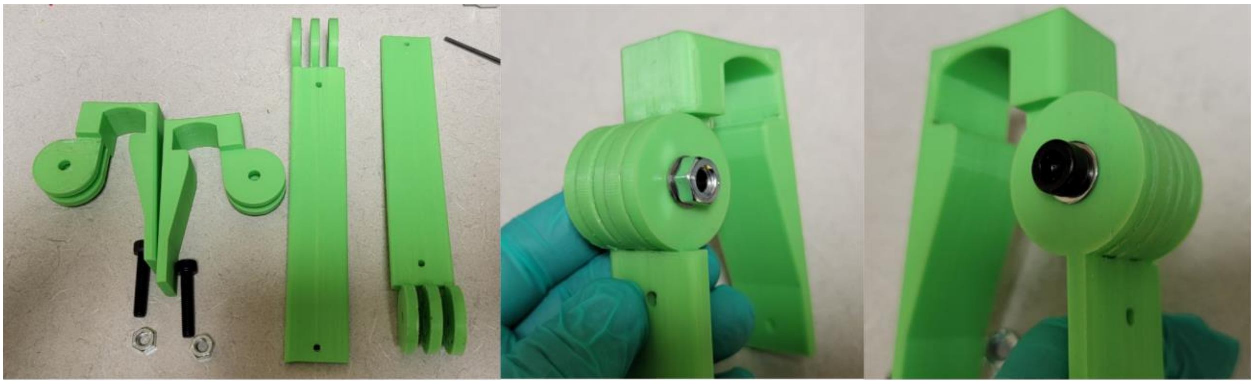

- Slide the teeth of one through the post and one cage snap piece. Once the pieces meet, slide a M6x30 with a washer and secure it with a washer and M6 nut.
- Repeat the process with the other through connector.

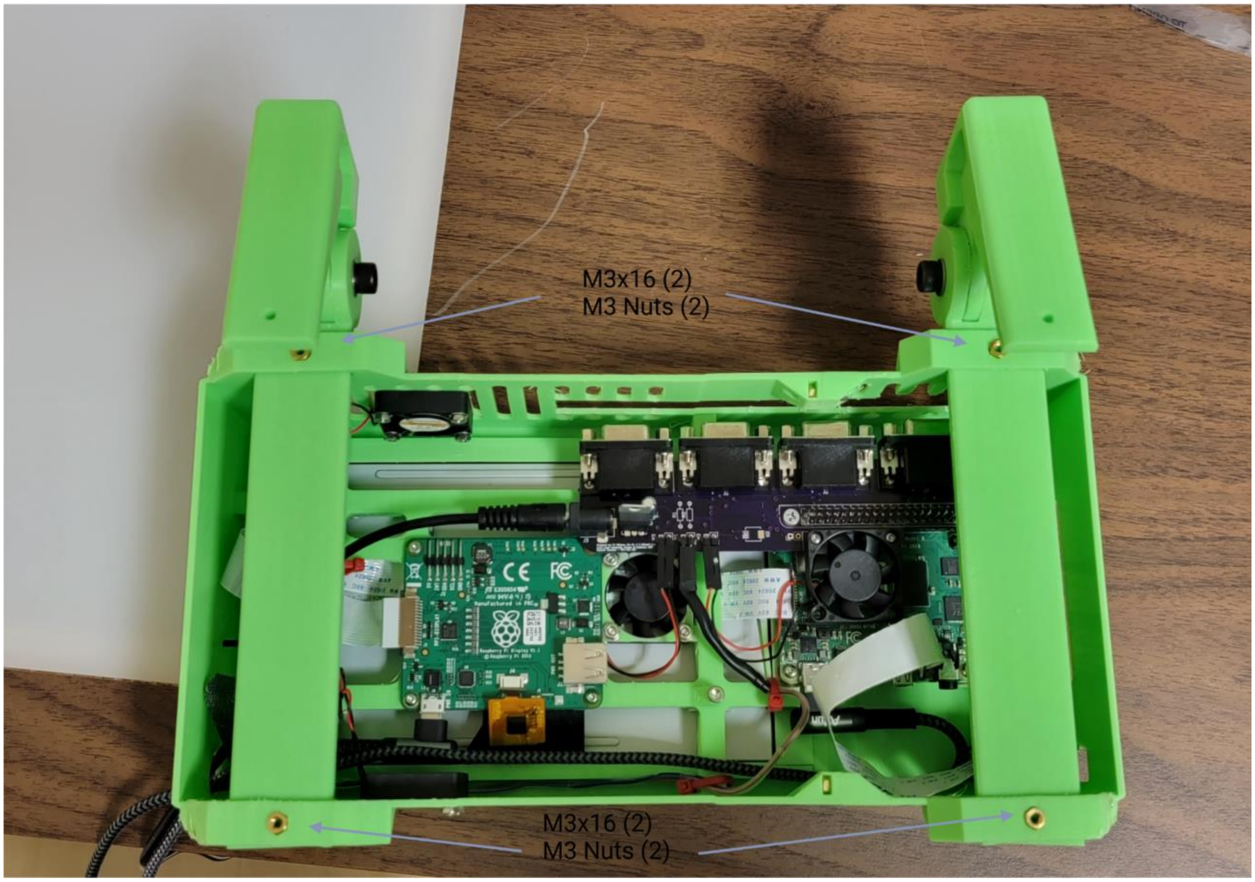

    - Next, slide the through posts into the support holes on the screen holder and secure using two M3x16 screws and M3 nuts, tighten to secure the holder arms to the screen holder. Repeat for the second arm, see below image for visual.

Step Five: Wiring the electronic components

- In order to install the components assembled in step three, you will need to ensure that you have all the necessary cables and cable management tools.
- The first step is to take the 0-24V power supply and all the power cables to do the installation.
- Wires can be installed bare or soldered directly onto the post of the power supply.
    - For a cleaner installation, we recommend using heat shrink wire connectors.
    - For the majority of the wires, using AWG 22 to 16 gauge is recommended. This will make installation of the wires onto the power supply post much easier
- After all the cables have been attached to the chamber, the next step is to make a hole for the power switch.
- Measure the dimension of your switch and then outline those dimensions onto your power supply box.
- Using a drill driver and a drill bit rated for metal, drill 4 holes for each of the four corners. (Do this in a well ventilated area).
- Next, take a multitool capable of cutting metal and connect the four holes along the lines you made when outlining the dimensions. (We used a Dewalt Oscillating Multi-tool w/ a Dewalt Titanium Metal Blade)
- Install the power switch and connect the cables to the power supply. The power supply box will have 4 holes which can be used to attach the power supply to the back of the box utilizing a screw.
    - Install the power supply in whatever orientation desired.
    - We recommend a vertical orientation although there is no correct or incorrect orientation.
    - Run the power cables through the punch holes and install cable management to keep the cables close to the chamber.

- Slide the Raspberry Pi and Pi holder onto the chamber and ensure that the power cables can reach all the boards and electronic components.

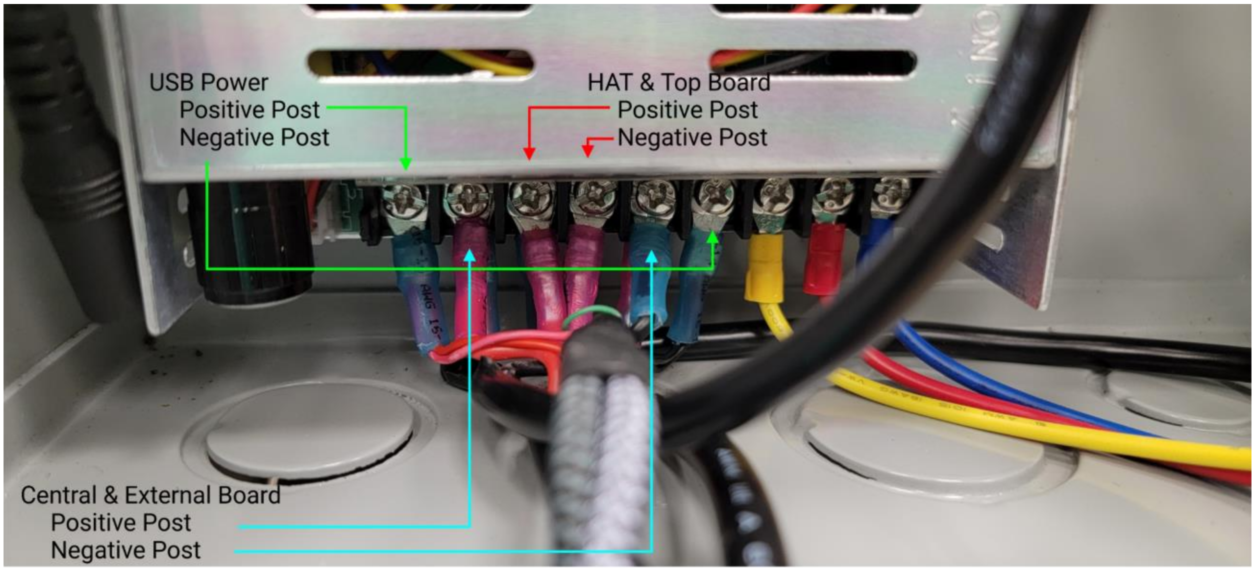

- Shut the power supply holder door and plug in the power. Turn on the power button and then open the door again. Using the adjustable, set the power supply for 5 V.
- Now we can attach the power cables to all the electronic devices. We recommend turning the power supply off before proceeding to prevent harm to you or the boards.
- Attach the VGA cables to the VGA connectors on the VGA HAT. Place the wires on the chamber with cable management hooks or straps until they are sitting at the locations where you want your sensor boards to be located.
- Connect the power cables to variable voltage power supply according to the following diagram. (ONLY ATTACH IF POWER HAS ALREADY BEEN SET TO 5 V. IF NOT, SET POWER SUPPLY TO 5 V BEFORE CONTINUING.)

Step Six: Installing the Raspbian Operating System

- Using the Raspberry Pi OS installer (https://www.raspberrypi.com/software/) and a blank microSD card (128 GB Recommended), write the raspbian OS onto the SD card. Under the "operating system" menu, select "Raspberry Pi OS (other)" and select "Raspberry Pi OS Full (32-bit)". Once the operating system is written to the SD card placed into Raspberry Pi.
- Once initial setup is completed go to terminal (Ctrl + T) and run the following codes:

```
sudo apt update

sudo apt full-upgrade
```

- Next step is to download WiringPi:

```
sudo apt-get update
```

git clone https://github.com/WiringPi/WiringPi

cd WiringPi

./build

- Once these applications have been downloaded check the version by running:

  gpio -v

  gpio readall

  If installed correctly you will see a readout of all the GPIOs in a table format (Figure 3)

- System settings also need to be modified. Go to the configuration settings by using the following code:

  sudo raspi-config

  Under "Interfacing Options" insure that camera, SSH, VNC, SPI, I2C, Serial, and Remote GPIO are all enabled

- System will require a reboot following these changes. The final items that need to be installed are all the Python packaged and Adafruit system requirements for CircuitPython. Tutorials and information can be found on Adafruit's website (https://learn.adafruit.com/welcome-to-circuitpython).

  sudo pip3 install adafruit-circuitpython-ads1x15

  sudo pip3 install adafruit-circuitpython-bme280

  sudo pip3 install adafruit-circuitpython-mcp9808

  sudo pip3 install adafruit-circuitpython-ccs811

  sudo pip3 install adafruit-circuitpython-bh1750

- Once all the background files have been downloaded the github library can be downloaded using

  wget https://github.com/drcgw/hypox-chamber.git

- Unpack the files by unzipping and move the *RunChamber.sh* file to the desktop.

Step Seven: Running System Checks

- Before running any of the Python codes or programs, a system check needs to be conducted to ensure that the Raspberry Pi is reading all the boards and sensors.
- First, connect all the boards to the Raspberry Pi VGA HAT to each of the breakout boards and power supply.
- Second, verify that all the sensor boards are getting power. You should be able to see green LEDs on each board, if one doesn't have a green LED illuminated then you need to check your soldering and connections.
- Finally, if all the visual inspections pass, then move on to checking the sensors through the terminal. Use the following code to detect what sensors are being detected:

```
i2cdetect -y 1
```

When running i2cdetect it will search the addresses and report which ones are returning a signal. Table 1 contains each sensor used and the corresponding addresses they can return. Additionally, Figure 4 shows the return of the code.

Table 1: List of sensors and their corresponding I2C addresses, originally obtained from Adafruit website.

Sensor	Available Addresses
MCP9808	0x18, 0x19, 0x1A, 0x1B, 0x1C, 0x1D, 0x1E & 0x1F
BME280	0x76 & 0x77
BME680	0x76 & 0x77
BH1750	0x23 & 0x5C
CCS811	0x5A & 0x5B
TSL2591	0x28 & 0x29
ADS1115	0x48, 0x49, 0x4A & 0x4B

Step Eight: Running an exposure

- The codes must be run through the terminal. The first step is to download the codes from GitHub and move the folder titled “Python_Codes” to documents (This may change in the future. Please see in the instructions on GitHub for most current versions instructions.
- For each exposure, three codes need to be run, the first is a BASH code to create the required directories for the chamber code to work properly.

- Move the BASH code to the Desktop or keep it in the home directory. In the terminal use the following commands to run the BASH script, this is for a file moved to the desktop.

  cd Desktop/

  chmod +x HypoxiaChamber.sh

  ls (The file should now appear green)

  ./HypoixiaChamber.sh

- The code will not return anything unless it fails, in which case an error message will be displayed.

- Now that the Directories have been created the following code will navigate to the Python code and run the code in the terminal:

  cd Documents/YYYY-MM-DD-Exposure/

  scripts YYYYMMDD-ExposureRecord.txt

  cd

  cd Documents/Python_Codes/

  ls (Please identify the most recent version of the chamber code)

  python3 YYYY-MM-DD-HypoxiaChamber_Version.py

  (Please check for the most recent version on GitHub

- The code will now run for a total of 7 days before automatically shutting down, if less time is needed the code can always be terminated by using Ctrl + Z.
- For customized timing or any other adjustments on how the program works, such as what boards are being used and what data will be recorded, edits can be made using a text editor like Geany, Nano or Anaconda.
